# Supplementary material for: Impact of health information technology optimization on clinical quality performance in health centers: A national cross-sectional study
Source: PLoS One. 2020 Jul 15;15(7):e0236019. doi: 10.1371/journal.pone.0236019 (PMC7363086; doi:10.1371/journal.pone.0236019)
Supplement: Attachment — Submitted filename: Reveiwer comment.doc [file pone.0236019.s001.doc]

Abstract

The aim of the study should be similar with the aim in the main text.

Please privde the references fort he alst paragrapg of the introduction.

Page 7 line 131 add mmHg after blood pressure value

Methods

Page 7- the last paragraph- The number of variables should continue with the number of 9 until 12 since you specified twelve variables.

Discussion section

The section should be supported with more up-to-date references. – especially 1st and 2nd paragraph needs more references
