# Supplementary material for: Impact of health information technology optimization on clinical quality performance in health centers: A national cross-sectional study
Source: PLoS One. 2020 Jul 15;15(7):e0236019. doi: 10.1371/journal.pone.0236019 (PMC7363086; doi:10.1371/journal.pone.0236019)
Supplement: Attachment — Submitted filename: Response to Reviewer.docx [file pone.0236019.s002.docx]

Response to Reviewer #1:

Reviewer #1-1 had stated a “No” response to Q4: Have the authors made all data underlying findings in their manuscript fully available?

Response: The authors had previously provided information in the manuscript submission questionnaire on how to request access to the Uniform Data System as follows: The Health Resources and Services Administration/Bureau of Primary Health Care (HRSA/BPHC)'s Health Center Program makes the Uniform Data Systems (UDS) available, for free, to the public in an electronic format. This action is being taken pursuant to the Freedom of Information Act (5 U.S.C. 552(a)(2)(D)) (FOIA). URL: https://www.hrsa.gov/foia/UDS-public-use.html)

Reviewer #1-2: On line 132 and in several other places you call the analysis bivariate. This is incorrect. Bivariate is a two level analysis, when you reference it is in terms of either the HC size (small, medium, large) or just being added without actual context. This should be removed from all text.

Response: Bivariate analysis is the analysis of two variables to study the empirical relationship between the variables.[1] Examples of the types of variable of interest could be binary, categorical, ordinal, nominal, and continuous. In Table 1, we analyzed practice size and each of the characteristics in the bivariate analysis. Practice size is a categorical variable with three categories of small, medium, and large.

Reviewer #1-3: within the results you list Statistical Significance, There has been a big shift in the literature to avoid this term and the ASA recommends to not use this term. Instead we should be thinking of clinical relevance. When reading your results I am not sure how much of the p-value is driven by sample size. Which a lot of that comes from presenting the information to allow the reader to interpret the findings accurately beyond your text.

Response: P-value is one of the tools in statistical hypothesis testing. Thus, we have chosen to report it in our manuscript. In response the Reviewer #1 comment 1-4, we have revised Table 4 to report the 95% confidence interval along with p-values to provide the reader with additional statistics in interpreting the findings.

With respect to the comment on clinical relevance, the study conducted a data analysis of the Uniform Data System (UDS), which is an administrative data set containing information on patient sociodemographic characteristics, primary care services provision, healthcare workforce, clinical quality measures (CQMs), and Meaningful Use (MU) attestation that are reported and aggregated at the health center organizational level as described in the Methods section. This study aims to examine the association between MU attainment supported by federal initiative and clinical quality performance at the health center organization level as stated in the Introduction section. This was not a clinical treatment study. No individual patient records are contained in UDS dataset. Thus, clinical relevance does not pertain to our study.

Reviewer #1-4: When reporting the model results you should be showing the Coefficient, the SE (Standard Error), 95% CI, Test statistic and p-value. Each of your models should report the overall model effect as well the R^2 associated with each. Also, it is typical to see all coefficients including intercept. You are trying to show a lot of information in the table so I can see the justification in only concentrating on the three variables of interest. In your response to showing SE, this is not acceptable. The reader needs to understand the variation about that change.

Response: Table 4 has been updated with coefficient, standard error, 95 CI, test statistic and p-value.

Reviewer #1-5: Also, you do not mention model assumptions, an appropriate model selection was not done and you do not mention interaction testing.

Response: We had conducted interaction testing, which demonstrated that interacting terms were not statistically significant. Thus, interaction terms were not retained in the regression model. [2] We have added a statement in the Methods section and reference in the revised manuscript.

References

1.	Tabachnick BG, Fidell LS. Using multivariate statistics (5th ed). Boston, MA: Allyn & Bacon/Pearson Education; 2007.

2.	Pagano M, Gauvreau K. Principles of Biostatistics 2nd Edition. Australia: Duxbury; 2000.
